# Complement activation in Ghanaian children with severe *Plasmodium falciparum *malaria

**DOI:** 10.1186/1475-2875-6-165

**Published:** 2007-12-17

**Authors:** Gideon K Helegbe, Bamenla Q Goka, Joergen AL Kurtzhals, Michael M Addae, Edwin Ollaga, John KA Tetteh, Daniel Dodoo, Michael F Ofori, George Obeng-Adjei, Kenji Hirayama, Gordon A Awandare, Bartholomew D Akanmori

**Affiliations:** 1 Department of Biochemistry and Molecular Medicine, SMHS, UDS, Tamale, Ghana; 2 Department of Child Health, Korle-Bu Teaching Hospital, Accra, Ghana; 3 Centre for Medical Parasitology, Department of Clinical Microbiology, Copenhagen University Hospital (Righospitalet), Copenhagen, Denmark; 4 Immunology Dept., NMIMR, College of Health Sciences, University of Ghana, Legon, Ghana; 5 Blood Bank, Korle-Bu Teaching Hospital, Accra, Ghana; 6 Department of Immunogenetics, Institute of Tropical Medicine (NEKKEN), Nagasaki University, Japan; 7 Biochemistry Department, University of Ghana, Legon, Ghana, and Division of Military Casualty Research, Department of Cellular Injury, Walter Reed Army Institute of Research, Silver Spring, MD, Ghana

## Abstract

**Background:**

Severe anaemia (SA), intravascular haemolysis (IVH) and respiratory distress (RD) are severe forms of *Plasmodium falciparum *malaria, with RD reported to be of prognostic importance in African children with malarial anaemia. Complement factors have been implicated in the mechanism leading to excess anaemia in acute *P. falciparum *infection.

**Methods:**

The direct Coombs test (DCT) and flow cytometry were used to investigate the mean levels of RBC-bound complement fragments (C3d and C3bαβ) and the regulatory proteins [complement receptor 1 (CD35) and decay accelerating factor (CD55)] in children with discrete clinical forms of *P. falciparum *malaria. The relationship between the findings and clinical parameters including coma, haemoglobin (Hb) levels and RD were investigated.

**Results:**

Of the 484 samples tested, 131(27%) were positive in DCT, out of which 115/131 (87.8%) were positive for C3d alone while 16/131 (12.2%) were positive for either IgG alone or both. 67.4% of the study population were below 5 years of age and DCT positivity was more common in this age group relative to children who were 5 years or older (Odds ratio, OR = 3.8; 95%CI, 2.2–6.7, p < 0.001). DCT correlated significantly with RD (β = -304, p = 0.006), but multiple regression analysis revealed that, Hb (β = -0.341, p = 0.012) and coma (β = -0.256, p = 0.034) were stronger predictors of RD than DCT (β = 0.228, p = 0.061). DCT was also not associated with IVH, p = 0.19, while spleen size was inversely correlated with Hb (r = -402, p = 0.001). Flow cytometry showed similar mean fluorescent intensity (MFI) values of CD35, CD55 and C3bαβ levels on the surfaces of RBC in patients and asymptomatic controls (AC). However, binding of C3bαβ correlated significantly with CD35 or CD55 (p < 0.001).

**Conclusion:**

These results suggest that complement activation contributed to anaemia in acute childhood *P. falciparum *malaria, possibly through induction of erythrophagocytosis and haemolysis. In contrast to other studies, this study did not find association between levels of the complement regulatory proteins, CD35 and CD55 and malarial anaemia. These findings suggest that complement activation could also be involved in the pathogenesis of RD but larger studies are needed to confirm this finding.

## Background

The mortality associated with malaria largely occurs in children as a result of complications, such as severe anaemia (SA), intravascular haemolysis (IVH), cerebral malaria (CM) and metabolic acidosis, clinically manifested as respiratory distress (RD) [1-5]. Sub-Saharan Africa accounts for 90% of the world's 300–500 million malaria cases and 1.5–2.7 million deaths annually [6]. A recent study has shown that in Ghana, the most common manifestations of severe malaria (SM) are SA (36.5%), followed by RD (24.4%) and CM (5.4%) [7]. There are no reports of the relative contribution of IVH to SM cases in Ghana.

IVH due to *P. falciparum *is a condition with high case-fatality if diagnosis and treatment are not optimal [8]. It is usually considered a rare complication of malaria in endemic areas, but recent studies have highlighted its importance [5,9]. Although it appears that the direct RBC destruction due to IVH is a minor contributor to malarial anaemia it is nonetheless strongly associated with erythrophagocytosis and with a poor prognosis [5,9]. Most studies of IVH in malaria have focussed on the influence of glucose 6-phosphate dehydrogenase (G6PD) [10-12], and the role played by antimalarial drugs such as chloroquine [13,14]. Mechanical trauma from a damaged endothelium, complement fixation and activation on the RBC surface, and infectious agents may cause direct membrane degradation and cell destruction [4].

It has been observed that the degree of red blood cell (RBC) breakdown during acute malaria cannot be explained solely by the direct destruction of RBC by malaria parasite schizogony [15]. Thus depletion of RBC is thought to be partly immune-mediated [15]. Infected erythrocytes bind to endothelial cells, and *P. falciparum *antigens known as erythrocyte membrane protein 1 (PfEMP1), inserted into the infected erythrocyte surface, mediate this interaction. It has been argued that these antigens are recognized by IgG subclasses that activate the classical complement pathway [16]. This pathway may also be triggered by binding of immune complexes or dead merozoites to the RBC surface [17]. As a result, monocytes, which have C3b and C3bαβ receptors on their surfaces, are activated to phagocytose the infected RBC. Thus, complement does not seem to kill parasites directly, but could play a role as an opsonin for neutrophils and macrophages [18]. In line with this, previous studies have shown that binding of complement factor C3d to RBC is common in childhood malaria, whereas IgG binding is rare [19].

A role for complement activation in RBC breakdown during malaria is supported by reports of positive DCT in patients with anaemia [20-23]. In addition, the balance between the beneficial immune activation functions of the complement cascade and its detrimental role in disease pathogenesis is maintained by a large number of regulatory proteins. Some of these include, complement receptor 1 (CD35), which binds C3b, and decay accelerating factor (CD55) and membrane attack complex inhibitor factor (CD59), which play a role in regulating haemolysis due to deposition of immune complexes on the surface of RBC [24]. Studies have shown that, deficiencies of these membrane-bound complement-regulatory proteins on infected and uninfected erythrocytes are associated with SM [25-28]. However, these studies did not use strictly defined patient categories and, in some cases malaria diagnosis was not confirmed in the control groups. In the present study, the role of complement activation in the pathogenesis of SM was investigated in a group of Ghanaian children presenting at hospital with strictly defined manifestations of malaria, including uncomplicated malaria (UM), SA, CM, IVH and RD. In addition, the binding of complement factors and the expression of complement regulatory proteins on RBC was investigated by flow cytometry. Given the high rate of mortality associated with RD in Ghanaian children [29], and the association between SA and RD [7], the relationship between complement activation and RD was also examined.

## Materials and methods

### Study design and patient population

Children between one and twelve years of age with severe forms of malaria as described previously [30], were consecutively recruited into an unmatched case control study in July-August, 2000 at the Emergency Unit of the Department of Child Health (DCH), Korle-Bu teaching Hospital, Accra, Ghana. Apparently healthy children, randomly selected from a database of children in the same age range from a nearby community, Dodowa were enrolled as control subjects (AC) [29,30]. Malaria transmission in the study area, a coastal savannah, is perennial with considerable seasonal variation, peaking during and immediately after the rains (May-October). Residents are estimated to receive about 20 infective bites per year and *P. falciparum *constitutes 98% of all infections [31]. The Scientific and Technical Committee of the Noguchi Memorial Institute for Medical Research and the Ethics and Protocol Review Committee of the University of Ghana Medical School, Korle Bu, Accra approved the study. All patients and control subjects were enrolled in the study only after signed, informed, parental consent was obtained.

### Clinical investigation and inclusion criteria

Patients with axillary temperature > 37.5°C of no other obvious cause than malaria were screened for inclusion by a project physician. Clinical parameters were documented on standard written forms. Spleen enlargement was assessed by palpation and quantified as cm below the left costal margin along the mid-clavicular line. Patients with *P. falciparum *parasitaemia ≥ 10,000 parasites/μL were enrolled into the study if they fell into one of the following patient categories [29]: SA: haemoglobin (Hb) < 5 g/dL, fully conscious with no episodes of severe bleeding, reported or observed convulsions; CM: Blantyre coma score ≤ 3 and duration of coma > 60 minutes, any Hb value and no record of recent severe head trauma and other cause of coma or neurological diseases; IVH: evidence of haemoglobinuria detected by the urine dipstick test (Roche Diagnostics Ltd, Great Britain) followed by microscopy as described elsewhere [21]; RD: rapid breathing plus one or more of the following: alar flare, chest recessions, use of accessory muscles for respiration, or abnormally deep breathing; UM: Hb > 8 g/dL, fully conscious, no other features of SM. Patients with Hb between 5 and 8 g/dL were only included if they fell into one or more of the patient categories: IVH, CM and RD.

### Exclusion criteria

Patients who were sickling positive were excluded. Other haemoglobinopathies were not taken into consideration. Patients were also excluded from the study based on evidence of other infectious disease such as typhoid or upper respiratory tract infections or any other identified cause of anaemia than malaria. Patients with a history of antimalarial treatment within 2 weeks prior to admission were excluded [13,14].

### Management of patients

Based on institutional practice at the time, all patients with UM were treated with a standard chloroquine regime at a total dose of 25 mg/kg body weight, given over three days. In the event of treatment failure, treatment with amodiaquine (10 mg/kg body weight per day, as single daily doses for three days) was instituted. All patients with SM were treated with either amodiaquine syrup via nasogastric tube at the same dosage as described, or intramuscular quinine dihydrochloride (10 mg/kg body weight, 8-hourly). Parenteral quinine was changed to syrup at the same dosage when patients regained full consciousness or after 72 hours (which ever was earlier), to complete a 7-day course. Patients with SA or RD were given humidified oxygen and children with Hb < 5 g/dL received blood transfusions [29].

### Blood sampling and laboratory analysis

Immediately after admission, 5 ml of venous blood was collected into EDTA tubes from all patients screened for inclusion. Haematological profile was determined using an 18-parameter, automatic haematology analyzer (Sysmex KX-21, Japan). Sickling test was done using the sodium metabisulphite test. Giemsa-stained thick and thin blood films were used for microscopic detection and identification of *Plasmodium *parasites. Parasites were counted against 300 WBC, and the value was converted into parasites per μL of peripheral blood, based on individual WBC count. Quality control (QC) was ensured by checking the autoanalyser daily and testing of 10% slides by second look. Errors were below 10% variation or disagreement and without systematic deviations.

### Direct Coombs' test

DCT was done using the method described by Goka *et al*. [21]. RBC suspensions were washed 4 times in comparatively large volumes (4 ml per wash) of 0.9% saline by centrifugation and reconstituted with saline to 5% PCV. One drop of this suspension was mixed with two drops of poly-antiglobulin sera (DIAGAST laboratories, Cedex, France), followed by centrifugation at 1,200 rpm for 1 minute. The samples were immediately inspected macroscopically for agglutination and negative or doubtful positive/weak results were re-examined by light microscopy. The positive samples were re-tested using mono-specific antiserum against IgG and C3d. Test results were graded as +4 (complete agglutination), +3 (several large agglutinates, few cells), +2 (large agglutinates in a sea of smaller clumps and free cells), +1 (many small agglutinates) or 0 (no agglutination). Positive and negative controls were run in parallel.

### Flow cytometry

Packed RBC were washed twice by centrifugation and resuspended in PBS (pH 7.2) to 2.0 × 10^7 ^cells/ml. The RBC suspension was stained with ethidium bromide (50 μg/ml final concentration), followed by surface staining with FITC-conjugated antibodies in the dark [32]. The following antibodies were used: Mouse IgG_1 _FITC (Pharmigen International, 33814X, San Diego, CA, USA); CD35 (Pharmigen, 30961A); IgG (Becton Dickinson, San Jose, CA, USA, 345140{5140}); Mouse IgG_2a _FITC (Pharmigen, 33034X); CD55 (Pharmigen, 33571A); Mouse IgG_1 _pure (DAKO, Glostrup, Denmark, X 0931); C3bαβ (Cymbus Biotechnology Ltd., Hampshire, UK, CBL 189 for α; CBL 190 for β); Rabbit IgG pure (Zymed Laboratories, INC., San Franscisco, California, USA, 81245172); C3d pure (DAKO, A 0063); C3d FITC (DAKO, F 0323) and controls (Goat anti-mouse FITC (DAKO, F0479); Swine anti-rabbit FITC (DAKO, F0054). After incubation, cells were washed twice with cellwash (Becton Dickinson) by centrifugation and samples were stored at 4°C in the dark till acquisition on a FACScan flow cytometer (Becton Dickinson, Japan). A minimum of 10,000 RBC were acquired and analysis was done by the CellQuest programme. The cut-off point was 10^1 ^on both axes.

### Statistical analysis

Statistical analysis was performed using SigmaStat software package (Jandel Scientific). Continuous variables were compared between groups using the student t-test or one way analysis of variance (ANOVA). For data that were not normally distributed and could not be normalised by logarithmic transformation a one-way ANOVA on ranks was used. Correlation between parameters was determined by Spearman rank order test. Proportions were compared using the Chi-square test, or Fisher's exact test. Variables that showed significant or near-significant differences between groups by univariate analysis or that were otherwise considered relevant for the study were entered in a conditional logistic regression analysis and in a multiple regression analysis. P-values < 0.05 were considered significant.

## Results

### Characteristics of the patient categories and controls

**Figure 1:**
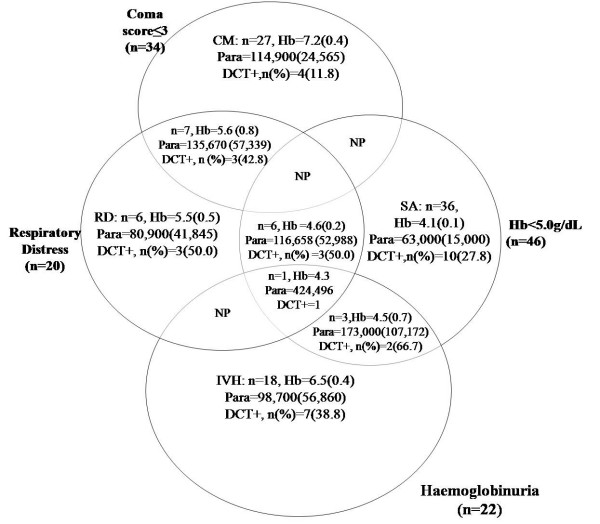
**Clinical categories of children with severe malaria**. Children admitted to the paediatric ward of the Korle-Bu teaching hospital, Accra, with severe malaria (SM). Symptoms of SM included coma (score ≤ 3 on the Blantyre coma scale), severe anaemia (SA) (haemoglobin [Hb] < 5.0 g/dL), intravascular haemoloysis (IVH, evidence of haemoglobinuria), and respiratory distress (RD). The number of children showing the various symptoms, as well as the mean (SEM) Hb (in g/dL) and parasitaemia (Para, in parasites per μL) plus prevalence of a direct Coombs' test (DCT) is shown. NP, no patient.

A total of 484 parasitaemic patients were screened for enrolment of which 87 patients with distinct clinical presentations of SM were included, 36 SA, 18 IVH, 27 CM and 6 RD. Their clinical characteristics together with those of UM patients and AC are summarised in Table 1. SA patients were younger (mean age = 2.5 yrs.) than the other categories of patients and the AC group (mean age 5.7 yrs.) (p < 0.001). SA had the lowest mean Hb (4.1 g/dL), followed by RD (5.5 g/dL) and IVH (6.5 g/dL). The highest mean Hb was found in AC (10.9 g/dL). All the patient categories had high parasitaemia whilst AC had the lowest parasite density (geometric mean, 1.4 × 10^3 ^parasites/μL). The geometric mean parasite densities of SA and UM cases (63.0 and 57.3 × 10^3^parasites/μL respectively) were significantly lower than those of the IVH, CM and RD cases (98.7 × 10^3^, 114.9 × 10^3 ^and 80.9 × 10^3 ^parasites/μL, respectively, p < 0.001). In order to study the effect of complement activation on RD, the data were re-analysed for all patients with SM, including those with overlapping clinical manifestations. The characteristics of these 104 children is summarised in Figure 1. Forty-six children had Hb < 5 g/dL, 34 had coma score ≤ 3, 22 had haemoglobinuria and 20 had RD.

**Table 1:** Demographic, clinical and laboratory characteristics of patients and healthy controls

**Characteristics**	**SA**	**IVH**	**CM**	**RD**	**UM**	**AC**	**p value**
n	36	18	27	6	21	19	-
Female sex, %	43.7	21.1	40.7	60.0	42.8	47.8	-
Mean Age, years (95% CI)	2.5** (1.8–3.1)	4.8 (3.1–6.6)	4.3 (3.3–5.3)	2.7** (2.0–5.5)	5.2 (3.6–6.8)	5.7 (4.4–7.1)	<0.001^b^
DCT Positive, n (%)	10* (27.8%)	7* (38.8%)	4 (11.8%)	3* 50.0%	2 (9.5%)	5.0* (26.3%)	<0.05^a^
Mean Hb, g/dL (95% CI)	4.1 (3.8–4.3)	6.5 (5.6–7.4)	7.2 (6.4–8.0)	5.5 (4.6–6.2)	9.2 (8.5–9.8)	10.9 (10.6–11.4)	-
Parasite density, geometric mean parasites × 10^3^/μL (95% CI)	63.0*** (34.3–94.1)	98.7 (47.6–284.5)	114.9 (67.4–162.6)	80.9 (12.1–256.9)	57.3*** (41.0–107.3)	1.4 (0–2.9)	<0.001^b^
Spleen size, cm (95% CI)	2.3 (1.5–3.0)	0.6(0–1.6)	0.7 (0.1–1.4)	ND	1.9 (0.6–3.2)	ND	-

Non-overlapping patient groups (see text). SA, severe anaemia (Hb < 5 g/dL). IVH, intravascular haemolysis with evidence of haemoglobinuria. CM, cerebral malaria (coma score ≤ 3). RD, respiratory distress. UM, uncomplicated malaria with Hb > 8 g/dL. AC, healthy, asymptomatic controls. DCT, direct Coombs' test. *Significantly higher than UM and CM. ** Significantly lower than CM, UM and AC. *** Significantly lower than IVH and CM. ND, not done.

a = Calculated using *X*^2^

b = Calculated using ANOVA

### Association of variables in children with severe malaria

Of all the parameters tested, only age and spleen size showed a significant correlation with Hb (Table 2). Age was positively correlated with Hb (r = 0.379, p = 0.002), and spleen size was inversely correlated to Hb (r = -0.402, p = 0.001).

**Table 2:** Correlation between variables in children with severe or uncomplicated malaria

	Hb		Spleen		Coma		Log para	
	
Variable	*r*	*p*	*r*	*p*	*r*	*p*	*r*	*p*
Age	**0.379**	**0.002**	-0.066	0.610	-0.142	0.265	-0.0618	0.630
Hb			**-0.402**	**0.001**	-0.201	0.114	-0.053	0.678
Spleen					0.270	0.032	-0.170	0.183
Coma							-0.208	0.102

Hb, haemoglobin. Spleen size, cm below left costal margin. Coma, score ≤ 3 on the Blantyre coma scale. Para, parasite (per μL). Patients analysed here are all the SM and UM cases (n = 125). Asymptomatic controls (AC) are not included.

### Clinical characteristics of DCT positive and negative patients and controls

Of all the patients screened (484), 27.0% (131) were DCT positive. Most of the sensitisation was due to C3d alone (87.8%), with a small proportion being ascribed to IgG alone (2.3%) or to both (9.9%). Children below 5 years formed 84.7% (111/131) of the DCT positives relative to those of 5 years and older, OR = 3.8 (95% CI, 2.2–6.7, p < 0.001). There was a high prevalence of DCT positive cases in the RD (50.0%), IVH (38.8%) and SA (27.8%) patients whereas UM and CM had 9.5% and 11.8% prevalence respectively. Surprisingly, 26.3% of the AC patients had positive DCT (Table 1). When overlapping cases were included in the analysis, RD patients still had the highest DCT positive rate with (50.0%, (10/20)), followed by IVH (45.5%, (10/22)); SA (34.8%, (16/46)) and CM (20.6%, (7/34)), Figure 1. Of all the children with severe forms of malaria (SM, n = 104), 33 (31.6%) were DCT positive (Table 3). There were no significant differences in age and parasitaemia between the DCT positive and the DCT negative patients of the SM (p > 0.05, Table 3), whereas the mean Hb was significantly lower in the DCT positive patients (p < 0.001). Although there was a trend toward higher prevalence of a positive DCT in RD, IVH, and SA, compared with CM, these associations were not statistically significant (Table 3).

**Table 3:** Clinical characteristics of all children with severe malaria grouped by DCT results

**Characteristics**	**DCT positive**	**DCT negative**	**P value**
n (%)	33 (31.6%)	71 (68.2%)	-
Age, years	3.3 (2.5–4.0)	4.0 (3.4–4.6)	0.11^b^
Hb, g/dL	5.0 (4.3–5.6)	6.6 (6.1–7.1)	<0.001^b^
Parasite density, × 10^3^/μL	125.6 (81.5–169.7)	139.7 (107.6–171.8)	0.33^b^
Mean spleen size, cm	2.1 (1.1–3.1)	1.2 (0.6–1.8)	0.09^b^
SA, n (%)	16 (48.5%)	30 (42.3%)	0.70^a^
CM, n (%)	7 (21.2%)	27 (38.0%)	0.15^a^
Haemoglobinuria, n (%)	10 (30.3%)	12 (16.9%)	0.19^a^
Mortality, n (%)	4 (12.1%)	15 (21.1%)	0.40^a^

Data are mean (95% confidence interval) unless otherwise indicated. Patients include all patients in Figure 1. Thus, the same patient can appear in several categories.

a = Calculated using *X*^2 ^or Fischer's exact test

b = Calculated using, student t-test

### Relationship between DCT result and RD

Out of 20 RD cases, 50.0% (10/20) were DCT positive compared with 27.4% (23/84) non RD cases, p = 0.092. Furthermore, the strength of the DCT result (0–4) correlated significantly with RD (standard coefficient β = -0.304, p = 0.006). Since children with RD appeared to be more likely to have DCT positive results compared to those without RD, the relationship between DCT result and RD was further investigated using multiple linear regression where the strength of the DCT result, Hb, coma score, and age were tested for their contribution to RD. In this model, Hb (β = -0.341, p = 0.012) and coma score (β = -0.256, p = 0.031, Table 4) emerged as the strongest predictors of RD compared with DCT grade (β = 0.228, p = 0.061) and age (β = 0.208 and p = 0.820). These results suggest that the association between RD and DCT may be related to the role of DCT in precipitating anaemia.

**Table 4:** Multiple regression analysis of variables predicting respiratory distress

Characteristics	Standard coefficient (β)	P-value
Age (years)	0.208	0.820
Coma score	-0.256	0.031
Hb (g/dL)	-0.341	0.012
DCT grade (0–4)	0.228	0.061

NB: Patients analysed here are all the SM and UM cases (n = 125). AC were not included.

### Relationship between opsonins (C3d and C3bαβ) and complement regulatory proteins (CD35 and CD55) in different patient categories

**Figure 2:**
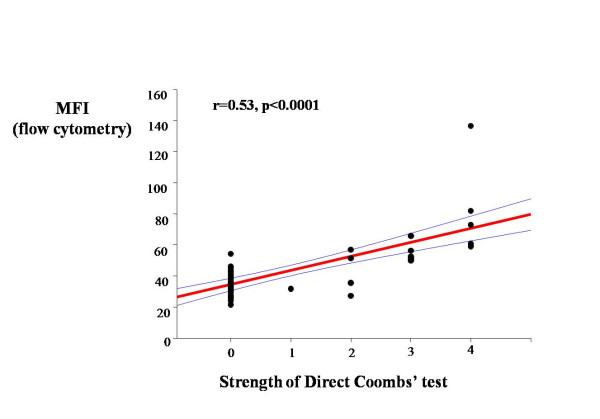
**DCT results compared with flow cytometry using anti-C3d**. RBC suspensions from 30 DCT positive and negative SM patients were stained with ethidium bromide and monoclonal antibodies to detect surface-bound C3d. Association between DCT results and flow cytometry results for C3d are shown by scatter plots with regression line. MFI, mean fluorescence intensity. DCT was graded as accordingly as +4 (complete agglutination), +3 (several large agglutinates, few cells), +2 (large agglutinates in a sea of smaller clumps and free cells), +1 (many small agglutinates), 0 (no sign of agglutination).

**Figure 3:**
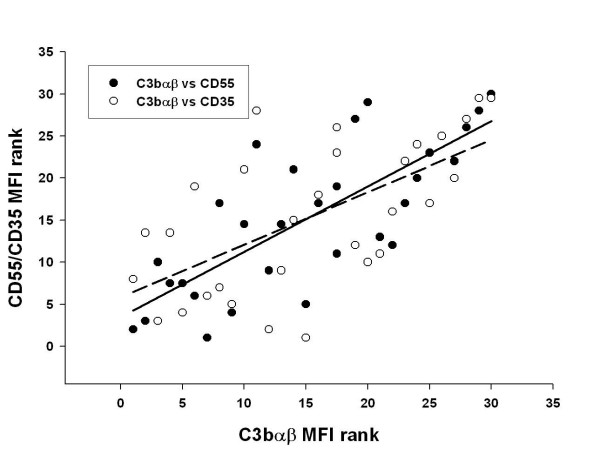
**Association of CD55 and CD35 with C3αβ in children with severe malaria**. Correlation between C3bαβ and complement regulatory proteins (CD35 (complement receptor type 1) and CD55 (decay accelerating factor)) measured by flow cytometry on surfaces of RBC from 30 children with SM. Solid line: C3bαβ vs CD55, r = 0.777, p < 0.001; dotted regression line: C3bαβ vs CD35, r = 0.624, p < 0.001. MFI, mean fluorescence intensity.

The association between the DCT method and flow cytometry was confirmed by a positive correlation between DCT grade and MFI after surface staining of RBC with anti-C3d (r = 0.53, p < 0.001, Figure 2). In order to study the influence of complement regulatory proteins on clinical outcome, binding of C3d and C3bαβ and expression of CD35 and CD55 on the RBC surface were measured by flow cytometry in a subset of the patients (Table 5). As shown in Table 5, there were no differences in any of the tested parameters between the main clinical groups (p > 0.7). The binding of C3bαβ correlated significantly with CD35 and CD55 (p < 0.001) in children with SM, Figure 3. There was no correlation between C3d and either CD35 or CD55 (p > 0.05, data not shown).

**Table 5:** MFI levels of complement factors and regulatory proteins by patient categories

Complement factors	Patient Categories and Controls					
	
	SA, n = 11	IVH, n = 8	CM, n = 11	UM, n = 10	AC, n = 11	P value^a^
CD35	2.10 (1.6–2.6)	2.09 (1.4–2.7)	2.08 (1.7–2.5)	1.85 (1.6–2.1)	1.97 (1.7–2.2)	0.88
CD55	2.05 (1.5–2.5)	1.76 (1.4–2.1)	1.94 (1.7–2.2)	1.85 (1.7–2.0)	1.98 (1.7–2.3)	0.81
C3bαβ	2.90 (0.7–5.1)	2.74 (1.1–4.5)	1.94 (1.1–2.8)	2.11 (1.4–2.8)	3.30 (1.2–5.4)	0.76

Data are represented as means (95% confidence intervals). CD35, Complement receptor type 1. CD55, Decay accelerating factor. C3bαβ, complement fragment from breakdown of C3b.

a = Calculated using one-way ANOVA.

## Discussion

Results from the present study show that infection with *P. falciparum *parasites stimulates complement activation, consistent with previous studies [21-23,33]. The complement activation was associated with reduced Hb-levels [21,22,24]. A central molecule in this association appears to be C3d that can opsonise the RBC for erythrophagocytosis. To a large extent, the erythrophagocytosis is mediated by macrophages, as indicated by increased levels of neopterin [29,30,34], which is a marker of macrophage activation [35]. The combination of activated macrophages and RBC opsonised by complement after activation will lead to increased erythrophagocytosis and decreased Hb as demonstrated in other studies [21,36]. This was reflected in an inverse correlation between spleen size and Hb and a trend toward larger spleen size in DCT positive than in DCT negative patients. Thus, phagocytosis of iRBC caused by macrophages in the spleen, enhanced by the opsonisation of RBC by C3d, may be one of the mechanisms by which Hb drops due to *P. falciparum *infection. However, a prospective study comparing DCT positive and negative patients in relation to drop in Hb after initiation of treatment would be relevant to confirm a causal relationship.

Complement activation could additionally contribute to malarial anaemia by direct lysis of infected RBC [5]. However, both this and a previous study [21] only showed a weak and insignificant association between DCT results and haemoglobinuria. Other factors apart from complement that may trigger IVH in malaria are drugs such as chloroquine [13,14], genetic factors [10,37] and autoimmunity [38]. Apart from excluding sickle cell genes, other factors were not controlled for in this study. However, the lack of association between DCT and IVH suggests that the main contribution of complement activation to the pathogenesis of malarial anaemia is opsonisation for erythrophagocytosis. The surprisingly high prevalence of DCT positives in the AC group indicates that a number of apparently healthy children may have an activated complement system. In a related study [39], it was observed that asymptomatic *P. falciparum *infection resulted in immune activation and anaemia in semi-immune children. The present study suggests that complement activation could contribute to the low Hb in the semi-immune children.

In the present study the DCT [40] was used alongside flow cytometry to determine the relationship between the levels of complement fragments (C3bαβ) and regulatory proteins (CD35 and CD 55). Other investigators have shown that activation and levels of regulatory proteins on surfaces of RBC are related to RBC loss in malaria [25,28]. In contrast, the present study did not find any relationship between anaemia and CD35 and CD55 levels. Unlike the current study, the previous studies did not use strictly defined patient categories and, in some cases malaria diagnosis was not confirmed. However, the discrepancy could also be due to differences in responses to *P. falciparum *of children from distinct geographical locations or exposed to varying levels of malaria transmission. There is a need to perform additional investigations of the role of complement regulatory proteins in the pathogenesis of malaria anaemia. The correlation between C3 fragment deposition and CD35 and CD55 levels reported here confirms results from previous studies [41], and suggests an adequate role of CD55 in regulating the extent of haemolysis in the studies patients.

Increased levels of inflammatory mediators have been shown to play a role in RD [29]. High levels of complement factors being reported here may implicate a synergistic role of these complement factors and inflammatory mediators in causing the RD due to *P. falciparum *infection. Such a synergistic interaction has been shown to take place during cardiac surgery with cardiopulmonary bypass (CBP) [42]. Similarly, in addition to complement activation during severe malaria infection, there is an imbalance between pro- and anti-inflammatory cytokines. Thus in children with SA complicated by RD, institution of effective treatment during the early phase of infection appears to be critical to survival [3,43]. The application of simple clinical and laboratory guidelines identifying children likely to develop SM and thus most in need for rapid interventions may thus improve survival and reduce unnecessary use of blood transfusion [3]. This study demonstrates that DCT can be used as a simple and reliable method of measuring complement activation in children with acute malaria and may be used to predict progression of UM to SA-RD and SA+RD. Prospective studies are needed to validate this diagnostic approach.

## Conclusion

The data presented here, supports a role for complement-mediated removal of RBC through erythrophagocytosis in the pathogenesis of malarial anaemia. In contrast to previous studies there was no relationship between the severity of anaemia and levels of complement receptor 1 or decay accelerating factor. Complement activation could also be involved in the pathogenesis of RD but larger studies are needed to confirm this finding. In addition the study demonstrates that DCT might be used as a simple method of predicting development of complications but this need to be studied prospectively in subsequent investigations.

## Competing interests

The author(s) declare that they have no competing interests.

## Authors' contributions

GKH designed the study, carried out laboratory work, data analyses and interpretation and drafted the manuscript. BQG and GOA assisted with design of study, selection, clinical examination and management of patients, as well as revision of manuscript for intellectual content. MMA and EO assisted with Coombs' test and data analysis. KH was involved with data analysis and extensive revision of manuscript for intellectual content. JKAT, DD and MFO designed the flow cytometry with GKH, acquired and analyzed the data. GAA was involved in data analyses, and preparation and revision of manuscript. JALK and BDA were involved in the design of experiment, data analysis and revision of manuscript for intellectual content.
